# Effects of Optimism on Work Satisfaction Among Nurses: A Mediation Model Through Work-Family Conflict

**DOI:** 10.3389/fpsyt.2021.779396

**Published:** 2021-11-25

**Authors:** Weiyu Zhang, Zhen Zheng, Romana Pylypchuk, Jinfeng Zhao, Kristin K. Sznajder, Can Cui, Xiaoshi Yang

**Affiliations:** ^1^Department of Social Medicine, College of Health Management, China Medical University, Shenyang, China; ^2^Department of Intensive Care Unit, Cancer Hospital of China Medical University, Liaoning Cancer Hospital and Institute, Shenyang, China; ^3^Faculty of Medical and Health Sciences, School of Population Health, The University of Auckland, Auckland, New Zealand; ^4^Department of Public Health, Pennsylvania State University College of Medicine, Hershey, PA, United States

**Keywords:** work satisfaction, optimism, work-family conflict, nurses, mental health

## Abstract

**Background:** Nurses are suffering from various stressors which adversely impact their work satisfaction and mental health. Research is scarce on optimism, one of the positive psychological resource which may reduce work-family conflict and improve work satisfaction.

**Objectives:** This study aims to assess work satisfaction among Chinese nurses and to observe and illustrate the relationships among optimism, work-family conflict, and work satisfaction.

**Methods:** This study was designed as a cross-sectional study with stratified sampling. From September 2019 to December 2020, a self-administered WeChat questionnaire was collected from 768 nurses online in China to evaluate the nurses' work satisfaction, optimism, and work-family conflict. Spearman correlation and hierarchical multiple regression analysis were applied to examine associated factors of work satisfaction. A structural equation model was employed to test the mediating effect of work-family conflict in the relationship between optimism and work satisfaction.

**Results:** Optimism were observed to have a positive correlation with work satisfaction while the correlation between work-family conflict and work satisfaction was observed to be negative. Optimism and work-family conflict explained 4.8 and 9.2% of the incremental variances of work satisfaction, respectively. Work-family conflict served as a mediator in the relationship between optimism and work satisfaction.

**Conclusions:** Nurses in China experienced high levels of work satisfaction. Optimism could increase the chance of higher work satisfaction while work-family conflict increased the risk of lower work satisfaction. Psychological interventions and improvement of working conditions are essential to relieve work-family conflicts and enhance work satisfaction.

## Introduction

The roles of nurses are more and more manifold and important in modern health care (1, 2). There are 4,445,047 certified nurses in China (3). With high demands of care responsibilities and rigorous training, stressors such as work-family conflict, nurse-patient conflict, long working hours, heavy workload, low promotion prospects, can adversely impact nurses' mental health, and work satisfaction (2–6).

Work satisfaction is the affective orientation that an employee holds to their work, comprising of two dimensions: intrinsic work satisfaction and extrinsic work satisfaction (7–11). The model of work environment satisfaction suggests that nurses' health and well-being are related to work satisfaction (12, 13). At the same time, dissatisfaction with work were found to be associated with increasing mental distress including anger, burnout, work stress, and frustration (10, 14–19). Furthermore, poor work satisfaction can result in decreasing health care quality (20). Therefore, the enhancement of work satisfaction of nurses is necessary to promote the health of both nurses and patients.

The proposed theoretical models explain the association between optimism and work satisfaction, by postulating that optimism could impact work satisfaction *via* various ways including improvements in work autonomy, enhancement in self-perception of health, promotion of perceived organizational support, etc. (21–24). Optimism is the positive and realistic expectation for success for the present and the future (25). Prior research has indicated the significant predictive power of optimism to work satisfaction and mental health (22, 26, 27). Related studies have found significant correlation between work satisfaction, optimism, and psychosomatic symptoms including fatigue, insomnia, breathing difficulties, etc. (28). Additionally, optimism can improve other positive work-related factors such as coping strategies, social resources, work engagement, work immersion, or organizational identification either directly or indirectly *via* mediation (29–33). In a word, promoting optimism among nurses can improve psychosomatic health and work satisfaction.

Besides optimism, it has been proved that work-family conflict has a close association with work satisfaction (34, 35). Work-family conflict refers to a psychological phenomenon of imbalance between work and family life (36). Studies have found that work-family conflict negatively associated with work satisfaction (37, 38). Work-family conflict can mediate the relationship between work satisfaction and work-satisfaction-related factors such as social support, professional values, and turnover intention (39–44). Work-family conflicts also exert effects on mental health, inducing burnout and depression (45, 46). A synthetic model of work-family demands and distress indicated that greater work-family conflict is predictive of depression (47). Thus, we hypothesized that work-family conflict might mediate the relationship between work satisfaction and other potential positive psychological factors, such as optimism. Efforts to mitigate work-family conflict may play a significant role in promoting work satisfaction among nurses.

Although increasing research has paid close attention to the mental health and work satisfaction among nurses, further studies are essential to explore the relationship among psychological factors, work-related factors, and work satisfaction with work-family conflict as a mediator (1, 48). Therefore, this study aimed to measure work satisfaction, optimism, and work-family conflict among Chinese nurses, and to investigate the potential relationships among them. The hypotheses of this study are as follows.

Hypothesis 1: Higher level of optimism is related with higher level of work satisfaction.
Hypothesis 2: Work-family conflict negatively impacts work satisfaction.
Hypothesis 3: Work-family conflict mediates the relationship between optimism and work satisfaction.

## Materials and Methods

### Participants, Procedure, and Ethics Statement

From September 2019 to December 2020, a multicenter cross-sectional survey designed with stratified sampling was carried out in Henan province and Liaoning province in China according to the distribution of population and nurses. With the preliminary communication with staff in the departments of medical administration, a total of 6 public hospitals (3 in Henan province and 3 in Liaoning province) were chosen as survey sites. Approximately 30% of the nurses working for clinical departments including internal medicine department, surgery department, emergency department, gynecology and pediatrics department, etc., were collected randomly as sample. A self-administered online questionnaire developed by Wenjuanxing, a widely used online questionnaire platform in China, was distributed *via* working group chat with a QR code. The inclusion criteria were as follows: Qualification Certificate of Specialty and Technology in Nursing obtained; at least 20 years of age; able to independently complete the online questionnaire in Chinese; able to provide signed informed consent and take part in the study voluntarily. The inform consent included the aims of this study, the further use of the data, and the strict protection of their privacy in this study. Only the participants checked the inform consent can they started the following online survey. Participants who met any of the criteria as follows were excluded: being diagnosed or treated with a severe mental illness (e.g., bipolar disorder, schizoaffective psychosis, paranoiac psychosis, etc.); suffering from conditions which prevented participation in this survey (e.g., visual impairment).

The questionnaire contained the Minnesota Satisfaction Questionnaire, Life Orientation Test- revised, Work-family Conflict Scale, and self-developed questions on demographic characteristics (49–51). The validated questionnaire took about 25 min to complete. A total of 768 nurses accomplished and submitted the questionnaire. The study complied with the Declaration of Helsinki as revised in 1989, and the protocol was authorized by the Ethics Committee of China Medical University (ID: 2020048).

### Baseline Characteristics of the Nurses

The collected baseline characteristics of the nurses included age, sex (male, female), marital status (married, other), education (junior college and below, bachelor degree, and above) monthly income [ ≤ 3,000 yuan ( ≤ US $464.4), 3,001–5,000 yuan (US $464.4 to $774.0), and >5,000 yuan (>US $774.0)]), and chronic disease including hypertension, coronary heart disease, diabetes, cancer, etc. (yes, no).

### Assessment of Work Satisfaction

Work satisfaction of nurses was measured with the Chinese version of the Minnesota Satisfaction Questionnaire (MSQ) (49, 50). The MSQ comprised of two subscales, including intrinsic work satisfaction and extrinsic work satisfaction. Participants rated each item of MSQ according to 5-point Likert-type scale (1 = very dissatisfied; 2 = dissatisfied; 3 = neutral; 4 = satisfied; 5 = very satisfied). The Cronbach's alpha for MSQ in this study was 0.977.

### Assessment of Optimism

Optimism of nurses was measured with the Chinese version of the Life Orientation Test- revised (LOT-R) (51). The scales contained 6 items with 5-point Likert-type scale (1 = strongly disagree; 2 = disagree; 3 = neutral; 4 = agree; 5 = strongly agree). The Cronbach's alpha for LOT-R in this study was 0.650.

### Assessment of Work-Family Conflict

Work-family conflict of nurses was evaluated with the Chinese version of the Work-family Conflict Scale (WFC) (52). The WFC contained two subscales, work interference with family conflict (WIF) and family interference with work conflict (FIW). Both of the subscales comprised of conflicts of time, stress and behaviors (53). Participants rated each item with 5-point Likert-type scale (1 = strongly disagree; 2 = disagree; 3 = neutral; 4 = agree; 5 = strongly agree). The Cronbach's alpha for WFC in this study was 0.954.

### Statistical Analyses

SPSS version 23.0 and Amos 17.0 statistical software for Windows (IBM Corporation) were employed to conduct the statistical analyses. The relationship between work satisfaction and demographic variables were explored with *t*-tests and one-way analysis of variance (ANOVA). The Spearman correlation analysis was conducted to test the correlations between work satisfaction, optimism, and work-family conflict. A hierarchical multiple regression (HMR) analysis was applied to explore associate factors of work satisfaction and their corresponding incremental contributions: Step 1: baseline characteristics of the nurses; Step 2: optimism of the nurses; Step 3: work-family conflict of the nurses. The work satisfaction scores, as the dependent variable, were continuous in HMR. The magnitude of associations between the dependent and independent variables were evaluated with standardized parameter estimates (standardized β). The mediating role of work-family conflict was explored using Structural Equation Models (SEM), with work satisfaction as the dependent variable, optimism as the independent variable, and work-family conflict as the mediator. A two-tailed *P* < 0.05 was considered to be statistically significant.

## Results

### Baseline Characteristics of the Participants

**Figure 1:**
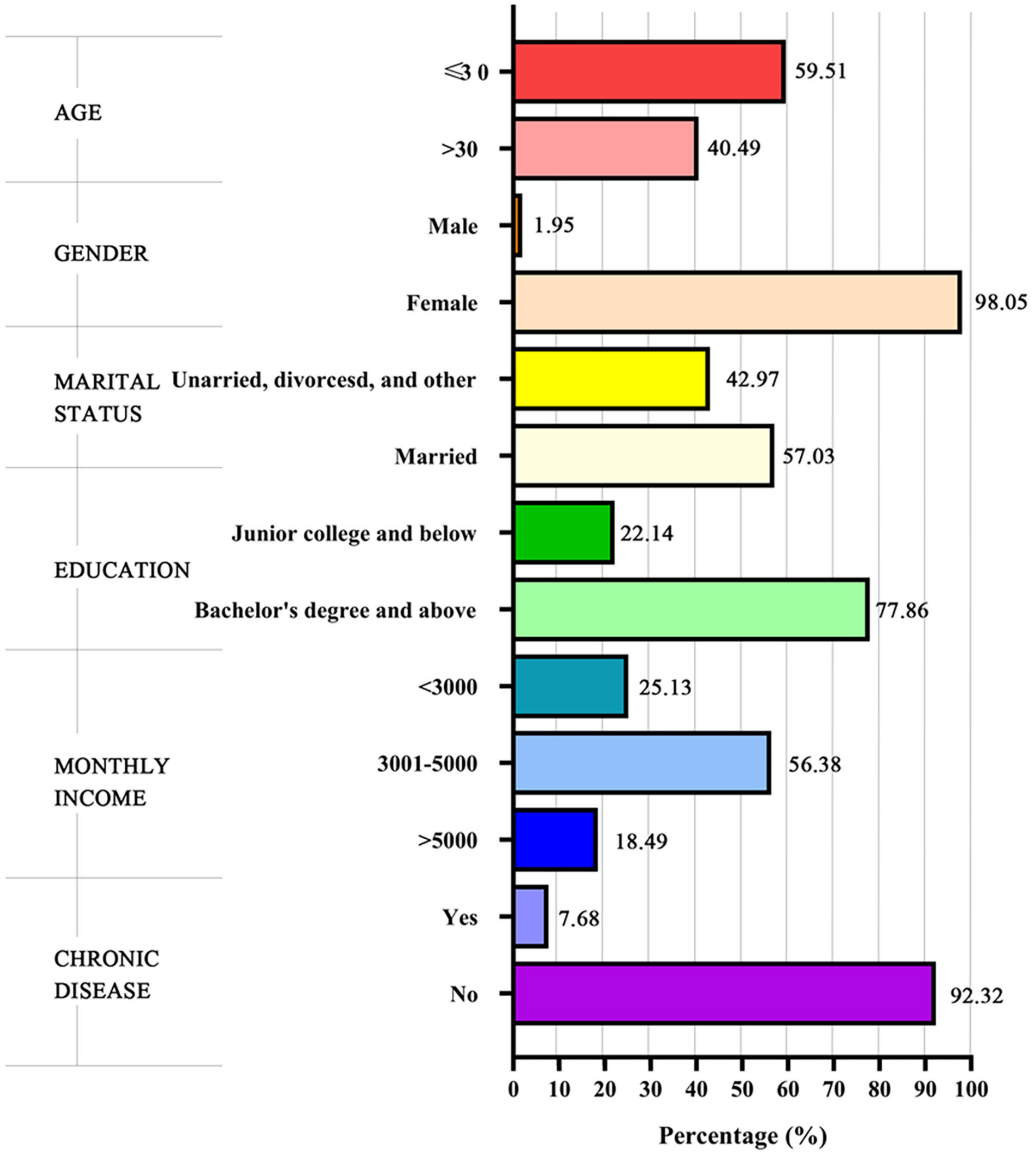
Baseline characteristics of the nurses.

The average score of the nurses' work satisfaction, optimism, and work-family conflict was 76.40 ± 16.26, 20.52 ± 3.50, and 51.31±14.91, respectively. The nurses' baseline characteristics and the distribution of work satisfaction are illustrated in Table 1 and Figure 1. A total of 768 nurses participated in the study. The mean age was 31 years and most of the participants were female (753, 98.05%). About half of the participants were married (438, 57.03%). More than three-quarters of the participants had a bachelor's degree or above (598, 77.86%). The results of the univariate analysis manifested that the work satisfaction scores were lower among participants who had a higher monthly income (*P* = 0.014). Participants who suffered from chronic diseases had lower work satisfaction (*P* = 0.001).

**Table 1:** Baseline characteristics of the distribution of the work satisfaction among nurses (*N* = 768).

**Variable**	***N* (%)**	**Work satisfaction (Mean ± SD)**	***P***
Age (years)			**0.753**
≤ 30	457(59.51)	76.25 ± 16.49	
>30	311(40.49)	76.62 ± 15.95	
Gender			**0.737**
Male	15(1.95)	75.00 ± 18.36	
Female	753(98.05)	76.43 ± 16.23	
Marriage			**0.178**
Unmarried, divorced, and other	330(42.97)	75.49 ± 17.22	
Married	438(57.03)	77.09 ± 15.49	
Education			**0.213**
Junior college and below	170(22.14)	77.77 ± 16.81	
Bachelor's degree and above	598(77.86)	76.01 ± 16.10	
Monthly income (¥^a^)			**0.014**
<3,000	193(25.13)	76.77 ± 16.39	
3,001–5,000	433(56.38)	77.40 ± 16.47	
>5,000	142(18.49)	72.85 ± 15.04	
Chronic disease			**0.001**
Yes	59(7.68)	69.47 ± 20.55	
No	709(92.32)	76.98 ± 15.74	

a *1¥ = US $0.15*.

*P: The possibility the data could have occurred under the null hypothesis*.

### Correlations Between Work Satisfaction, Optimism, and Work-Family Conflict

Table 2 shows that work satisfaction was significantly associated with optimism and work-family conflict (*P* < 0.01). Optimism had a positive correlation with work satisfaction (*P* < 0.01), whereas work-family conflict had a negative correlation with both work satisfaction and optimism (*P* < 0.01).

**Table 2:** The correlations among work satisfaction, optimism and work-family conflict.

	**1**	**2**	**3**	**4**
1.Work satisfaction	1			
2.Age	−0.001	1		
3.Optimism	0.274^**^	0.033	1	
4.Work-family conflict	−0.352^**^	0.018	−0.384^**^	1

** *Significant at the 0.01 level (two-tailed)*.

### Factors Associated With Work Satisfaction

**Figure 2:**
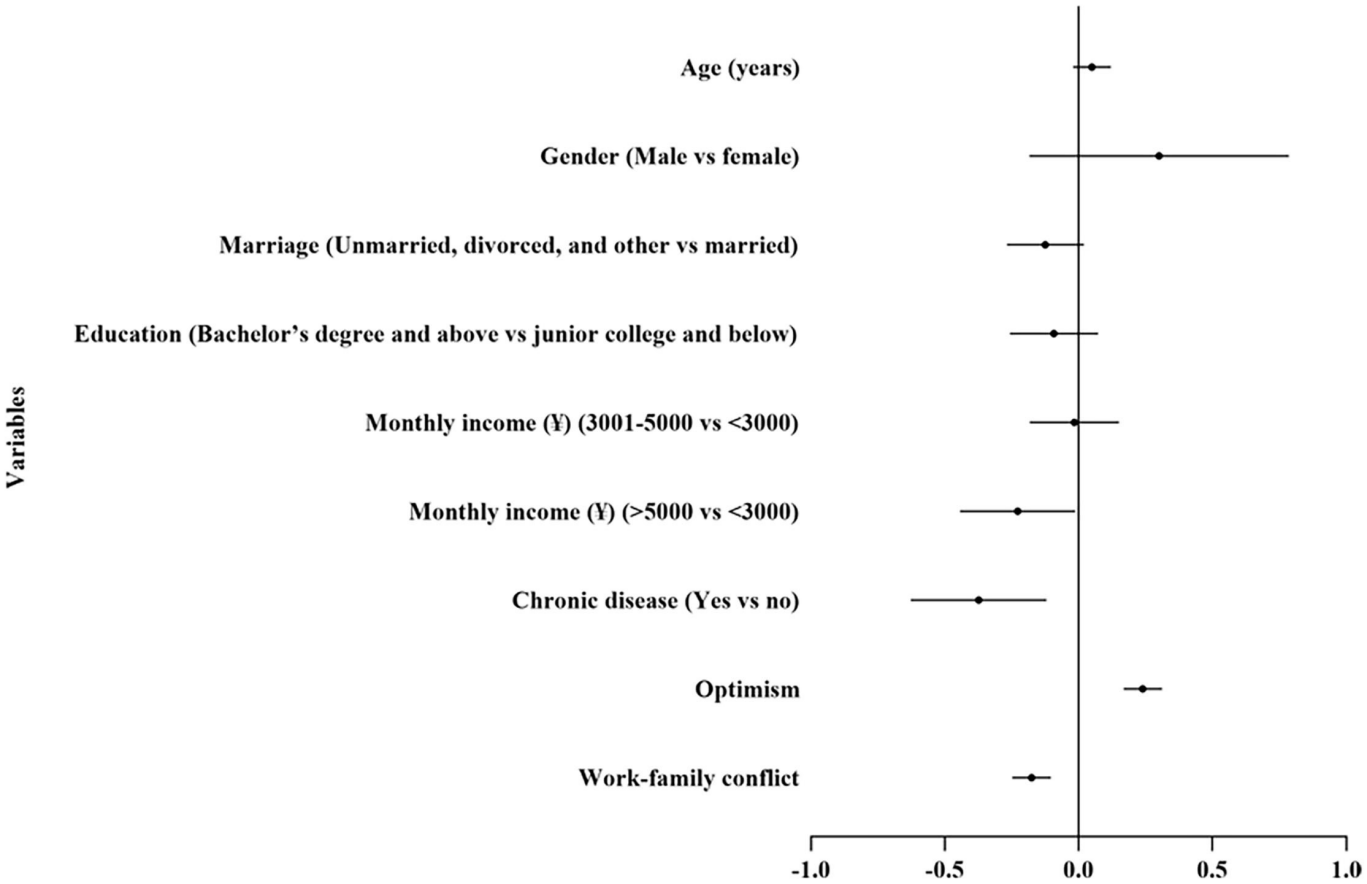
Forest plot of associate factors of work satisfaction.

The final results of the HMR models for work satisfaction among the nurses are presented in Table 3. A total of 15.1% of the variance was explained by the final model. Demographic characteristics, optimism, and work-family conflict explained 3.3, 4.8, and 9.2% of the incremental variances, respectively. According to the forest plot (Figure 2), optimism had a positive association with work satisfaction (*P* < 0.001). At the same time, work-family conflict had a negative association with work satisfaction (*P* < 0.001). Higher monthly income (>5,000 yuan) (*P* = 0.038) and suffering from a chronic disease (*P* = 0.004) were negatively associated with work satisfaction.

**Table 3:** The hierarchical multiple regression analysis of work satisfaction of the nurses.

**Variable**	**Model 1**			**Model 2**			**Model 3**		
	**β**	**Standardized β**	**95%CI**	**β**	**Standardized β**	**95%CI**	**β**	**Standardized β**	**95%CI**
**Demographic characteristics of the nurses**									
Age (years)	0.062	0.062	−0.013 to 0.137	0.067	0.067	−0.005 to 0.138	0.050	0.050	−0.021 to 0.120
Gender (Male vs. female)	0.102	0.014	−0.412 to 0.617	0.271	0.038	−0.220 to 0.762	0.301	0.042	−0.184 to 0.785
Marriage (Unmarried, divorced, and other vs. married)	−0.100	−0.049	−0.252 to 0.052	−0.094	−0.047	−0.239 to 0.051	−0.124	−0.062	−0.268 to 0.019
Education (Bachelor's degree and above vs. junior college and below)	−0.093	−0.039	−0.268 to 0.083	−0.088	−0.036	−0.255 to 0.079	−0.092	−0.038	−0.257 to 0.072
Monthly income (¥^a^)									
3,001–5,000 vs. <3,000	0.034	0.017	−0.143 to 0.212	−0.008	−0.004	−0.176 to 0.161	−0.016	−0.008	−0.182 to 0.151
>5,000 vs. <3,000	−0.221	−0.086	−0.450 to 0.007	−0.257^*^	−0.100^*^	−0.475 to −0.039	−0.228^*^	−0.089^*^	−0.443 to −0.013
Chronic disease (Yes vs. no)	−0.474^**^	−0.126^**^	−0.744 to −0.204	−0.407^**^	−0.108^**^	−0.664 to −0.15	−0.374^**^	−0.100^**^	−0.628–0.120
Optimism				0.307^**^	0.307^**^	0.240 to 0.374	0.240^**^	0.240^**^	0.168–0.3110
**Work-family conflict**							−0.176^**^	−0.176^**^	−0.248 to −0.104
*R* ^2^		**0.033**			**0.126**			**0.151**	
Adjusted *R*^2^		**0.024**			**0.116**			**0.141**	
Δ*R*^2^		**0.033**			**0.093**			**0.026**	

* *P < 0.05*,

** *P < 0.01*.

a *1 ¥ = US $0.15*.

*R^2^: A statistical measure in a regression model that determines the proportion of variance*.

*Adjusted R^2^: A modified version of R^2^ that accounts for predictors that are not significant in a regression model*.

*ΔR^2^: The change in R^2^ between two equations*.

### Work-Family Conflict as a Mediator Between Work Satisfaction and Optimism

**Figure 3:**
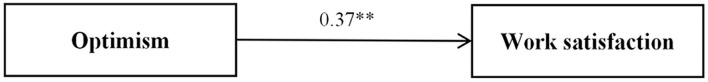
Standardized solution for the structural equation model of optimism and work satisfaction. **P < 0.01.

**Figure 4:**
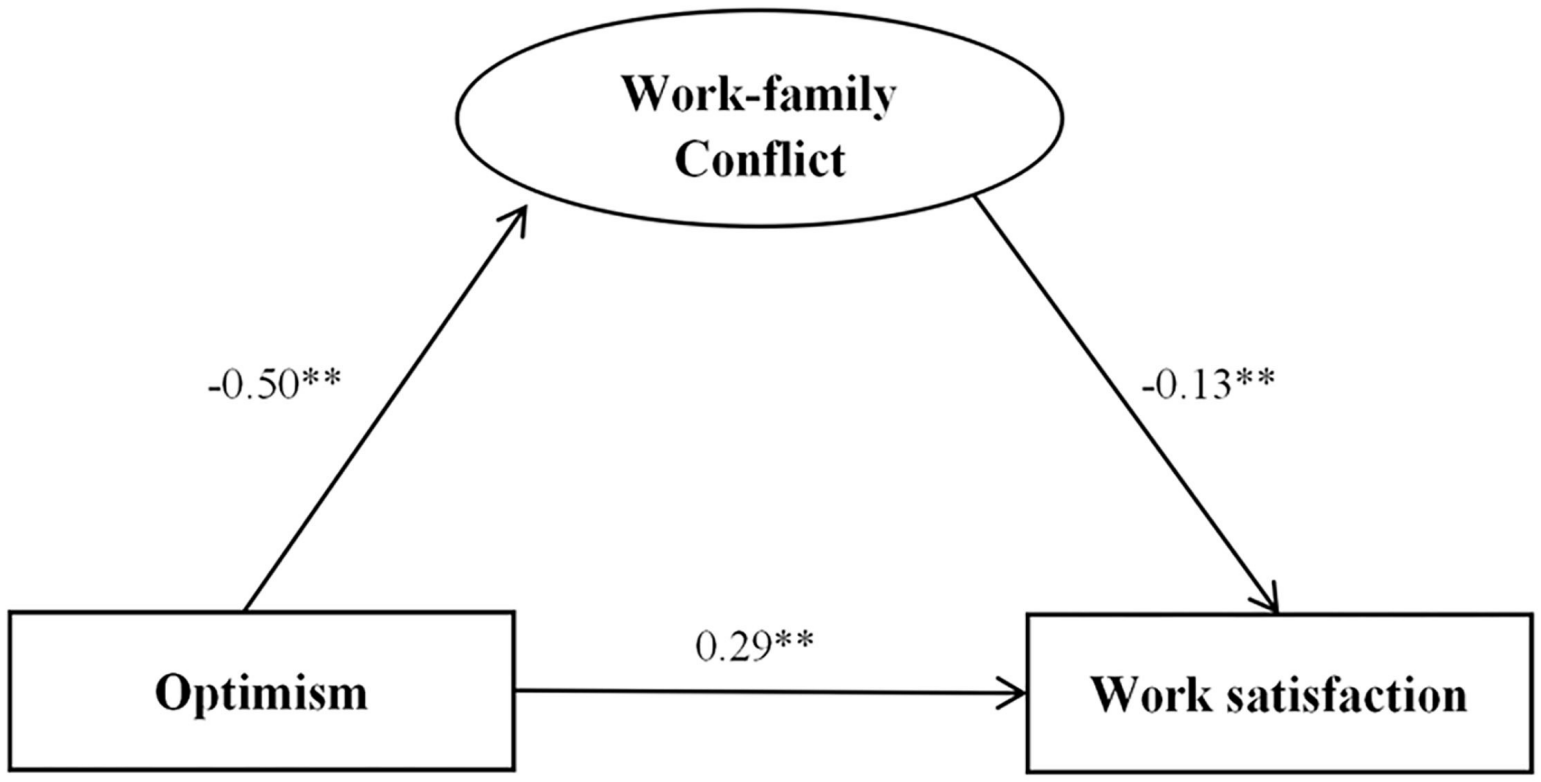
Standardized solution for the structural equation model of work-family conflict, optimism, and work satisfaction. **P < 0.01.

Table 4 present the outcomes of SEM analysis. The path coefficients and standardized solution for SEM are illustrated in Figures 3, 4. Optimism influenced work satisfaction directly (c = 0.37, *P* < 0.001). Optimism was positively associated with the work satisfaction (*P* < 0.001). The model fit indices were ideal (χ^2^/df = 2.636 <5, GFI = 0.990 > 0.9, AGFI = 0.970 > 0.9, CFI = 0.993 > 0.9, TLI = 0.984 > 0.9, RMSEA = 0.046 <0.05) (Figure 3). Work-family conflict had a significant association with work satisfaction (*P* < 0.001).

**Table 4:** The path coefficients of the mediation model.

	**B**	**β**	**S.E**.	**C.R**.	***P***
Work-family conflict←Optimism	−2.302	−0.50	0.239	−9.650	<0.001
Work satisfaction←Work-family conflict	−0.283	−0.13	0.090	−3.148	<0.001
Work satisfaction←Optimism	2.804	0.29	0.501	5.602	<0.001

*B, the unstandardized path coefficient; β, the standardized path coefficient; S.E., the standard error; C.R., the critical ratio*.

The path coefficient of optimism with work satisfaction decreased significantly when work-family conflict was considered as a mediator (c = 0.29, *P* < 0.001) (Figure 4). The direct path coefficient of optimism on work satisfaction was reduced or lost statistical significance after including the mediator, work-family conflict, which also suggested that the mediation effect existed. Work-family conflict mediated the association between optimism and work satisfaction (a^*^b = 0.08, BCa95%CI: 0.113–1.329, Percentile 95%CI: 0.111–1.326). The model fit indices were also ideal (χ^2^/df = 2.779 <5, GFI = 0.975 > 0.9, AGFI = 0.947 > 0.9, CFI = 0.986 > 0.9, TLI = 0.974 > 0.9, RMSEA=0.048 <0.05).

## Discussion

### Principal Findings

The average score of the nurses' work satisfaction, optimism and work-family conflict was 76.40 ± 16.26, 20.52 ± 3.50, and 51.31 ± 14.91 in this study, respectively. The correlations among work satisfaction, optimism, and work-family conflict were significant. Besides, this study observed that work-family conflict partially mediated the association between optimism and work satisfaction. According to other research which used the MSQ to measure work satisfaction of nurses, the level of work satisfaction among nurses was slightly higher than that of physicians in China (62.2–71.6) (24, 54, 55). Although there were studies which have explored the relationship among work satisfaction, optimism, and work-family conflict, the research investigated the role of work-family conflict in the relationship between optimism and work satisfaction remains scarce (56, 57).

In this study, optimism had a positive association with work satisfaction, which is consistent with former research (23, 24). According to the HMR analysis, optimism was a protective factor for work satisfaction, indicating that optimism may increase the chances of higher work satisfaction. As positive psychological capital, optimism can directly influence work autonomy, which can improve work satisfaction among health care workers (21). Therefore, through the promotion of optimism, nurses may enhance their abilities to cope with work tasks and improve their work satisfaction (22).

A negative correlation was observed between optimism and work-family conflict. Optimism can alleviate the negative outcomes of work-family conflict such as reduced work performance, decreased life and work satisfaction, and increased work pressure (55, 58–60). A study by Hao et al. found that optimism partially mediated the relationship between work-family conflict and depression (56). While the research focused on the relationship between optimism and work-family conflict is scarce, the role of optimism in work-family conflict still deserves exploration to improve work satisfaction and mental health.

Additionally, in this study, a negative correlation was observed between work-family conflict and work satisfaction, which is in agreement with previous research (61, 62). HMR analysis indicated that work-family conflict was a risk factor for poor work satisfaction. Additionally, work-family conflict was observed to have a mediating effect in the relationship between optimism and work satisfaction. When work-family conflict was considered as a mediator, the positive effect of optimism on work satisfaction was negatively impacted, indicating that optimism can prompt work satisfaction by effective control and decrease in work-family conflict. Working in an environment which requires a high level of ethics, responsibility, training, and specialization, work-family conflict is an adverse factor that intensifies the negative experience in nurses' career life (42, 63–66). Previous studies have found that work-family conflict could serve as a mediator in the relationship between work satisfaction and doctor-patient relationship, turnover intention, role commitment, and work involvement (42, 63–66). This study indicates that the reduction or elimination of work-family conflict is urgently needed to improve nurses' work satisfaction, which is also necessary for better patient outcomes.

Optimism and work-family conflict are critical factors linked with work satisfaction among nurses. Meanwhile, work-family conflict serves as a mediator between optimism and work satisfaction. Therefore, a supportive work environment should be developed with effective measures to set reduction of work-family conflict, enhance nurses' psychological health, and promote their work satisfaction; ultimately improving quality of care.

### Limitations

Several limitations should be acknowledged in this study. First, since the survey was conducted among nurses in two provinces in China, Henan and Liaoning, the generalizability to other populations is limited. Second, since this study was carried out from September 2019 to December 2020, the outcomes of this study may be limited by the outbreak of Coronavirus Disease-19 (COVID-19) pandemic and other unmeasured confounders. Third, selection bias cannot be ruled out because the survey was conducted online and the participants were smart phone users only. Finally, the baseline characteristics did not include professional factors such as year of experience, specialty and professional title, which should be added in the future studies.

## Conclusions

Nurses in China experience a high level of work satisfaction. Optimism increases work satisfaction while work-family conflict lowers work satisfaction and serves as a mediator between optimism and work satisfaction. The findings manifest that measures such as effective psychological interventions to increase optimism and to reduce work-family conflict should be taken to protect nurses' mental health, enhance nurse's work satisfaction, and importantly, to increase the quality of medical care for patients.

## Data Availability Statement

The raw data supporting the conclusions of this article will be made available by the authors, without undue reservation.

## Ethics Statement

The studies involving human participants were reviewed and approved by the Ethics Committee of China Medical University. The patients/participants provided their written informed consent to participate in this study.

## Author Contributions

WZ and ZZ analyzed the data and drafted and revised the manuscript. RP, JZ, and KS revised the manuscript. CC contributed to the acquisition and interpretation of data. XY was responsible for the conception and design and the revision of the manuscript. All authors contributed to the article and approved the submitted version.

## Funding

This work was supported by the fellowship of China Postdoctoral Science Foundation (2020M681022).

## Conflict of Interest

The authors declare that the research was conducted in the absence of any commercial or financial relationships that could be construed as a potential conflict of interest.

## Publisher's Note

All claims expressed in this article are solely those of the authors and do not necessarily represent those of their affiliated organizations, or those of the publisher, the editors and the reviewers. Any product that may be evaluated in this article, or claim that may be made by its manufacturer, is not guaranteed or endorsed by the publisher.
